# Cytokinesis During the First Division of a Mouse Embryo

**DOI:** 10.3389/fcell.2021.815599

**Published:** 2022-02-01

**Authors:** Xiao-Yan Fan, Xing-Hua Wang, Feng-Yun Xie, Jun-Yu Ma, Xiang-Hong Ou, Shi-Ming Luo

**Affiliations:** ^1^ Fertility Preservation Lab, Reproductive Medicine Center, Guangdong Second Provincial General Hospital, Guangzhou, China; ^2^ Guangdong-Hong Kong Metabolism & Reproduction Joint Laboratory, Guangdong Second Provincial General Hospital, Guangzhou, China

**Keywords:** meiosis, mitosis, zygote, microtubules, microfilaments, cytokinesis, cell cycle, intercellular bridge

## Abstract

Cell division consists of nuclear division (mitosis for somatic cells and meiosis for germ cells) and cytoplasmic division (cytokinesis). Embryonic developments are highly programmed, and thus, each cellular event during early embryo development is stable. For mouse embryos, the first time of mitosis is completed about 22 h after fertilization. However, it remains unclear when the embryo completes its first cytokinesis. Here, we microinjected only one cell in the 2-cell stage mouse embryos with mRNA, which encodes green fluorescence protein (GFP). By monitoring the GFP protein transport dynamics between the two cells, we demonstrated that the first time of cytokinesis in mouse embryos is completed about 15 h after mitosis, namely 37 h after fertilization. In addition, our results indicate that the cytoplasmic protein transport between daughter cells is very effective, which relies on microtubules instead of microfilaments in 2-cell mouse embryos. These results should enrich people’s understanding of the first cell division and cytoskeleton in mouse embryos and then learn more about the mechanisms of early embryo development in mammals.

## Introduction

Cell division is the foundation of life. Unicellular life directly utilizes it to produce offspring, and multicellular life utilizes it to generate new cells to grow bigger. In eukaryotic cells, cell division usually consists of two steps. One is genome separation, which is called mitosis or meiosis, depending on somatic or germ cells. The other one is cytokinesis, which includes the separation of cytoplasm and other organelles, such as mitochondria, endoplasmic reticulum (ER), ribosomes, etc. (Glotzer 2001; Eggert et al., 2006; Green et al., 2012). People usually indicate mitosis or meiosis as the cell division, including nuclear and cytoplastic separation. However, mitosis or meiosis only is the nuclear separation at first (Glotzer 2001; Eggert et al., 2006; Ruchaud et al., 2007), so we insist on this statement and thus distinguish them from cytokinesis in this study. Indeed, nuclear and cytoplastic separation is not strictly distinguished. Cytokinesis is also involved in genome division, and the absence of cytokinesis may lead the cells to be syncytial or death (Li 2007; Haglund et al., 2011). Therefore, cytokinesis plays a vital role in cell proliferation and differentiation as the final step of cell division.

Embryo development is highly programmed, and each cellular event occurs step by step. The first mitosis is completed about 22–24 h after fertilization, dependent on the mouse strain, and the zygote morphologically divides into a 2-cell embryo simultaneously (Nagy et al., 2003; Gray et al., 2004). However, it is still unclear when the first cytokinesis of cell division is completed.

Cytokinesis usually begins from anaphase and continues through telophase. Therefore, it occurs concurrently with mitosis or meiosis (Pollard 2010). Briefly, the cell cortex senses the spindle formation during early anaphase and produces an actomyosin ring at the equatorial position. Then the actomyosin ring contracts and turns into a cleavage furrow, which finally partitions the cytoplasm into the two daughter cells (Barr and Gruneberg 2007; Pollard 2010; Green et al., 2012). Notably, before the last step of cytokinesis that abscission occurs, the cytoplasm in the two daughter cells often transfers to each other by an intercellular bridge (Greenbaum et al., 2011; Haglund et al., 2011). So, monitoring the cytoplasmic protein transfer between the two daughter cells can determine the time point of completion of cytokinesis.

In this study, after the zygote morphologically divides into a 2-cell embryo, we microinjected only one cell with two types of mRNA. One expressed green fluorescence proteins (GFP), and the other expressed mitochondrial localized red fluorescence proteins (RFP). Thus, we could determine the time point of completion of the first cytokinesis in mouse embryos by monitoring the protein transfer dynamics in the two daughter cells. Furthermore, we artificially divided the two daughter cells at different time points through micromanipulation, thereby investigating the possible function of cytoplasmic communication that relies on the intercellular bridge. The results showed that the embryonic development and cell synchronization of the 2-cell separated embryos were similar to the control groups.

## Materials and Methods

### Plasmids Construction and mRNA Preparation

Two types of mRNA were used in this study. One is GFP, and the other is mitochondrial localized RFP. The GFP plasmid was constructed from a GFP-labeled actin plasmid by removing the actin sequence utilizing a cloning recombination kit (ClonExpress II One Step Cloning Kit, C112, Vazyme Biotech Co. Ltd., Nanjing, China). The mitochondrial localized RFP was constructed by replacing the lysosome sequence with a mitochondrial transmembrane peptide in a lysosome-localized RFP plasmid. The mitochondrial transmembrane peptide sequence was from a human CDGSH iron-sulfur domain-containing protein (9–36 amino acids, MVRVEWIAAVTIAAGTAAIGYLAYKRFYV) and was synthesized by Suzhou Genewiz Biotechnology Co., Ltd. Furthermore, we inserted a T7 promotor (TAA​TAC​GAC​TCA​CTA​TAG) to the GFP and mitochondrial localized RFP plasmids. We obtained the mRNA by utilizing an RNA transcription kit (HiScribeTM T7 ARCA mRNA Kit (with tailing), E2065S, New England Biolabs, United States). The GFP-labeled actin and RFP-labeled lysosome plasmids were gifts from Addgene (mEmerald-Actin-C-18, #53978; mApple-Lysosomes-20, #54921).

### Mouse Embryo Preparation and Culture

All the animal experiments in this study were carried out following the guidelines of the Animal Experiment Standard of Guangdong Second Provincial General Hospital. Briefly, 8 weeks old ICR mice were purchased from Beijing HFK Bioscience Co., Ltd and bred in a 12/12 h light/dark period. One week later, female mice were intraperitoneally injected with 10 IU of pregnant horse serum gonadotropin (PMSG) and 10 IU of human chorionic gonadotropin (hCG) at an interval of 48 h (Ningbo Animal Hormone Factory, Ningbo, China) for superovulation. After being mated with male mice, the copulatory plug was checked on the following day to confirm the successful mating. Then, the mated female mice were sacrificed by cervical dislocation, and the zygotes were collected with M2 medium and cultured in KSOM droplets covered by paraffin oil at 37 °C in a 5% CO2 atmosphere for development.

### Microinjection and Manipulation

The development of zygotes was monitored with a time-lapse microscope (Cyto-MINI, Guangzhou Sipu Photoelectric Technology Co. LTD, China). With piezo-assisted micromanipulation (Eppendorf PiezoXpert, Eppendorf AG, Hamburg, Germany) equipped with a straight microneedle (∼0.5 μm inner diameter), the collected 2-cell embryos were microinjected with mRNA in one random daughter cell at various time points after the formation of morphological 2-cell (Chen et al., 2021). In order to study the role of microtubules and microfilaments in the cytoplasmic communication, embryos cultured in the KSOM with 5 μg/ml cytochalasin B (Sangon Biotech (Shanghai) Co., Ltd., A606580-0005) or 10 μg/ml nocodazole (Sangon Biotech (Shanghai) Co., Ltd., A606391-0010) after mRNA injection, respectively. The possible function of cytoplasmic communication was studied by destroying the intercellular bridge artificially at various times after the zygotes were divided into 2-cell embryos. Briefly, the 2-cell stage embryos were cut a small opening in the zona pellucida with a laser system (Hamilton Thorne Instruments Biosciences, Beverly, Mass, United States), and then the two daughter cells were completely separated firstly and reaggregated immediately by an NT-88-V3 micromanipulator System equipped with a holding pipette and a straight tip pipette (20–25 μm inner diameter).

### Fluorescence Imaging

To dynamically monitor the cytoplasmic communication between the two daughter cells, the 2-cell embryos were first microinjected with mRNA expressing GFP and mitochondria localized RFP and then were imaged with a high-speed spinning disk confocal microscopy (Andor Dragonfly 200, Andor Technology, Belfast, United Kingdom) per hour. The GFP fluorescence intensity was obtained and calculated by ImageJ. The ratio of GFP intensity in the two daughter cells was used as the indicator to analyze the dynamics of cytoplasmic communication. Briefly, images selected from the GFP fluorescence channel were first turned to gray-white style, and the fluorescence intensity from each daughter cell and the areas outside the embryos as background were obtained. Then, after subtracting the background fluorescence intensity, the ratio of GFP fluorescence intensity in each pair of daughter cells was calculated.

### Data Analysis

Each experiment was repeated more than three times, and the samples were more than ten embryos in this study. Statistical results were analyzed by Origin2019 and expressed as mean ± SEM. and analyzed by two-tailed Student’s t-test. Statistically significant values of *p* > 0.05 and *p* < 0.05 are indicated as n.s. and asterisks^∗^, respectively.

## Results

### Cytoplasmic Green Fluorescence Protein Transmitted Between the Two Daughter Cells in 2-Cell Mouse Embryos

**FIGURE 1:**
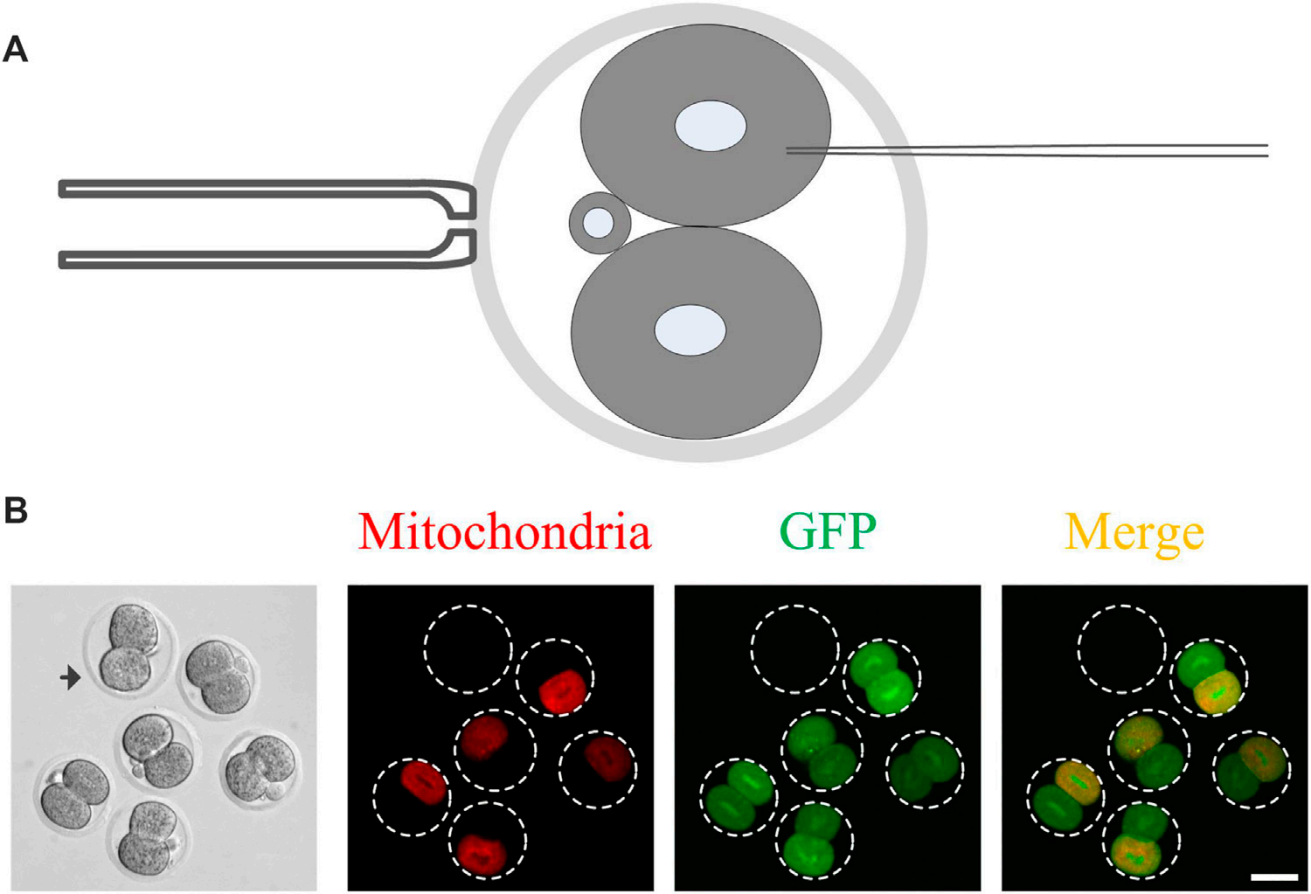
The cytoplasmic GFP but not the RFP-labeled mitochondria can be transported between the daughter cells in 2-cell mouse embryos. **(A)** A schematic of mRNA microinjection. The 2-cell embryo is held vertically by a holding pipette so that the microinjection pipette can completely avoid another daughter cell. Only one random daughter cell was microinjected with mRNA; **(B)** Representative images of the cytoplasmic communication. The cytoplasmic GFP but not the RFP-labeled mitochondria were transported from one daughter cell to another daughter cell. After 4 h of the mouse embryo, which morphologically divided into two cells, the mRNA expressing GFP and mitochondrial localized RFP was microinjected into it and images were captured 15 h later. The arrowhead indicates the control embryo, which was added in the samples to eliminate autofluorescence leading to false-positive results with weak fluorescence. Scale bar = 50 μm.

The last step of cytokinesis is to abscise an intercellular bridge between the two daughter cells, and thus, the two daughter cells are physically and utterly separate from each other (Mierzwa and Gerlich 2014). Before the abscission occurs, cytoplasm in the daughter cells may communicate by the intercellular bridge (Greenbaum, Iwamori, Buchold and Matzuk 2011; Green et al., 2012). So the closure of the intercellular communication between daughter cells could indicate the completion of cytokinesis. Whether cytoplasmic communication exists in the 2-cell mouse embryos is not clear. To clarify this issue, after 4 h of the mouse embryos divided into 2-cell stage, mRNA expressing GFP and mitochondrial localized RFP were microinjected into any one of the daughter cells (Figure 1A). Subsequently, the distribution of GFP and RFP were imaged under a fluorescence microscope at 15 h after microinjection. As shown in Figure 1B, the cytoplasmic GFP is evenly distributed in the two daughter cells, while the mitochondrial localized RFP only appeared in the daughter cell, which was injected with the mRNA. These results indicate that the intercellular bridge can transport the cytoplasmic localized proteins while not the organelles between the two daughter cells. Thus, it is possible to clarify the completion time of cytokinesis by monitoring the GFP transportation in 2-cell mouse embryos.

### The Dynamics of Green Fluorescence Protein Transportation Between the Two Daughter Cells and the Completion Time of Cytokinesis in 2-cell Mouse Embryos

**FIGURE 2:**
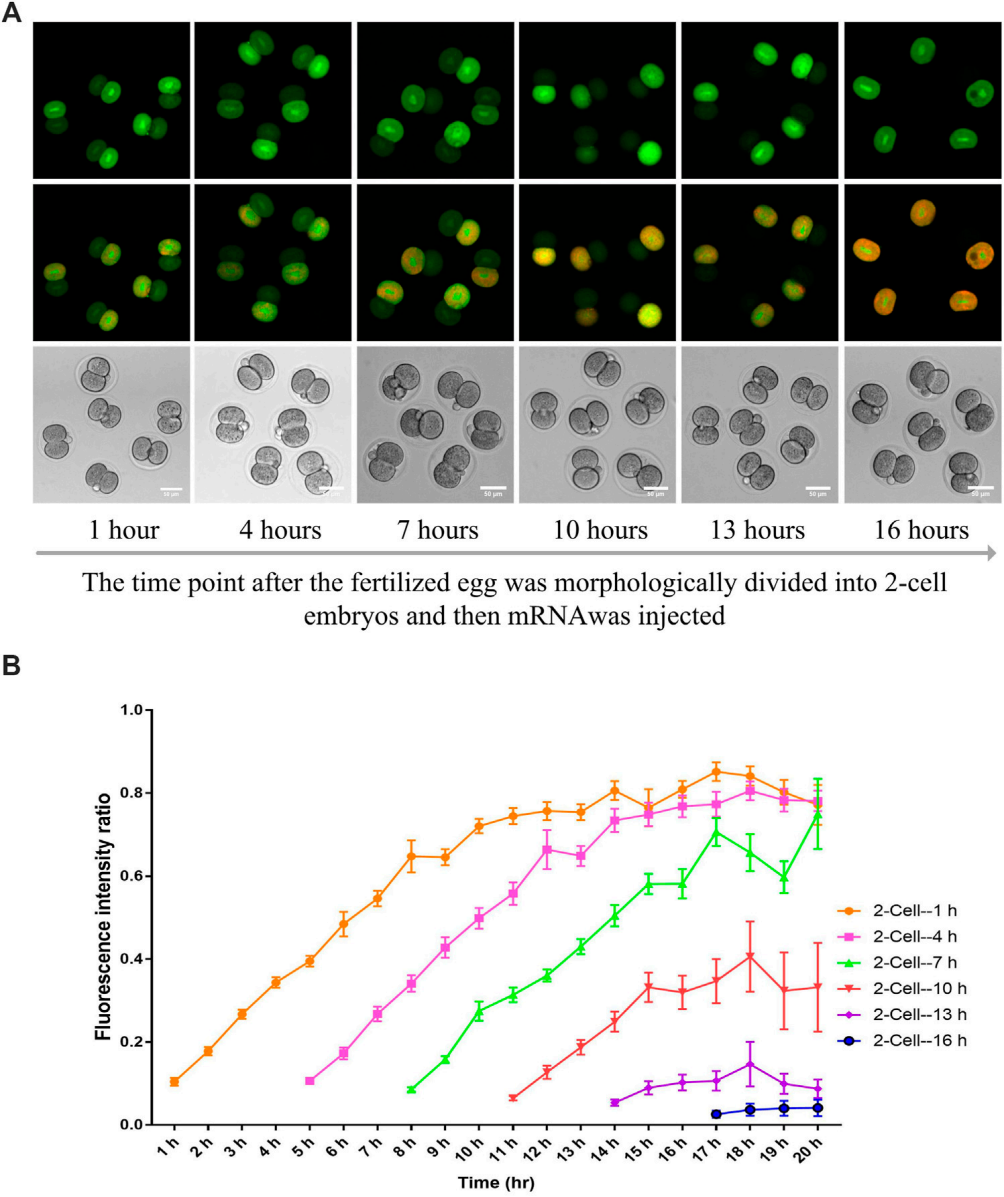
The analysis of GFP transport dynamics between the two daughter cells in 2-cell mouse embryos. **(A)** Representative images of 2-cell embryos injected with mRNA at different times and imaged 4 h later. **(B)** The ratio of GFP intensity between the GFP mRNA non-injected daughter cell and the GFP mRNA-injected daughter cell. One daughter cell from the 2-cell embryo at different time points was microinjected with GFP-expressing mRNA and imaged every hour later. The time points include 1, 4, 7, 10, 13 and 16 h after the fertilized egg morphologically divided into two cells. Each experiment was replicated three times, and *n* = 13–15 for each group. The data are expressed as mean ± SEM.

To determine the completion time of the first cytokinesis in mouse embryos, we monitored the dynamics of GFP transportation between the two daughter cells in 2-cell mouse embryos. Briefly, 1 h after the zygotes were morphologically divided as 2-cell embryos, GFP mRNA was microinjected into any one of the two daughter cells every 3 h, namely 1, 4, 7, 10, 13 and 16 h. Subsequently, the GFP was imaged every hour until 20 h. The GFP fluorescence intensity was examined, and its ratio between the two daughter cells was calculated (non-injected one/injected one). Results shown in Figure 2 indicate that based on the time points that the GFP mRNA was injected into the daughter cell (1, 4, 7, 10, 13 and 16 h after the zygotes morphologically divided as 2-cell embryos), the cytoplasmic GFP could transfer to the other daughter cell for about 11, 8, 5, 2 and 0 h, respectively. After 16 h, no cytoplasmic GFP transportation is found. Therefore, the cytoplasmic GFP transportation could continue about 15 h (4 + 11, 7 + 8, 10 + 5, 13 + 2 h) after the formation of morphological 2-cell. Hence, the cytokinesis is completed about 15 h after the zygotes morphologically enter the 2-cell stage. Furthermore, in the 1-, 4- and 7-h groups, the GFP linearly transferred to the other daughter cell for about 8 h and subsequently entered into a steady-state. In these groups, the GFP fluorescence ratio between the two daughter cells finally reached nearly 0.8. This result indicates that the intercellular communication is highly effective and non-directional in the early 2-cell mouse embryos (If the cytoplasmic transportation is direction-dependent, it should be that half of the embryos cannot transport GFP, while all the more than 100 embryos examined in our study transported the GFP to the other daughter cell). Moreover, the cytoplasmic protein transportation is concentration-dependent (If the cytoplasmic transportation is not concentration-dependent, it should be in some embryos the GFP fluorescence ratio between the two daughter cells more than 1:1.).

### The Role of Cytoplasmic Communication in Development and Cell Synchronization

**FIGURE 3:**
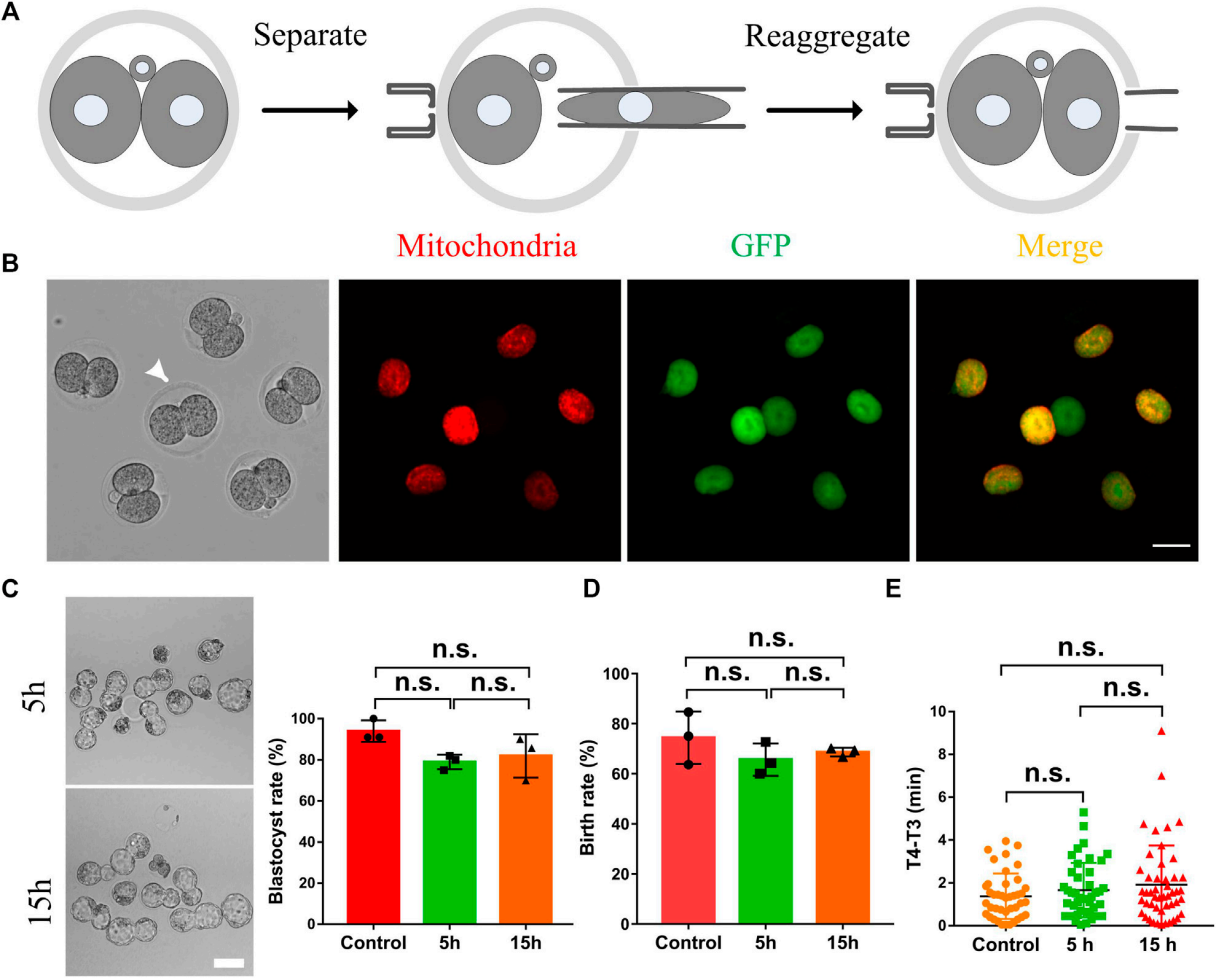
The effects of the intercellular bridge on embryo development and cell synchronization. **(A)** The schematic diagram separates and re-aggregates daughter cells in a 2-cell embryo. The two daughter cells were first completely separated by micromanipulation, and then they were immediately put back to reaggregate the embryos. **(B)** Representative fluorescence images of the re-aggregated embryos. Arrows indicate embryos that have not been separated and re-aggregated as a control. Scale bar = 50 μm. **(C)** Representative images and statistics of blastocysts developed from 2-cell embryos which were separated and reassembled at five and 15 h after the formation of 2-cell embryos. Scale bar = 100 μm. **(D)** Statistical analysis of the birth rate generated from 2-cell embryos which were separated and reassembled at five and 15 h after the formation of 2-cell embryos. **(E)** The time interval (T4-T3) between the 3-cell stage and the 4-cell stage of the embryos which were conducted with a separation and reaggregation at five and 15 h after the formation of 2-cell embryos. Each experiment was replicated three times, *n* = 30–34 for **(C)**, 36–40 for **(D)**, and 46–52 for **(E)**. The data are expressed as mean ± SEM and analyzed by two-tailed Student’s t-test. Statistically significant values of *p* > 0.05 indicate as no significance (n.s).

It is well known that intercellular communication plays essential roles in the cellular synchronization and differentiation in the female and male germline formation in metazoans, from insects to humans, and other syncytial cells such as early *Drosophila* embryos (Haglund et al., 2011). Therefore, we supposed the cytoplasmic communication in 2-cell mouse embryos might also play essential roles in cellular synchronization and embryo development. As shown in Figure 3A, 5 h or 15 h after the morphological division of the fertilized egg, the two daughter cells were separated by micromanipulation, and then immediately, they were put back into the same place. The cytoplasmic GFP transportation was examined to exclude the possibility that the cytoplasmic communication may be re-established after the two daughter cells reaggregated. The results indicated the GFP fluorescence was only observed in the daughter cell injected with mRNA (Figure 3B). This study selected the time interval (T4-T3) between the 3-cell stage and the 4-cell stage to represent the synchronization. Results shown in Figures 3C–E indicate that terminating the cytoplasmic communication beforehand has little effect on the blastula development, birth rate and cell synchronization.

### The Role of Microtubules and Microfilaments in the Cytoplasmic Communication

**FIGURE 4:**
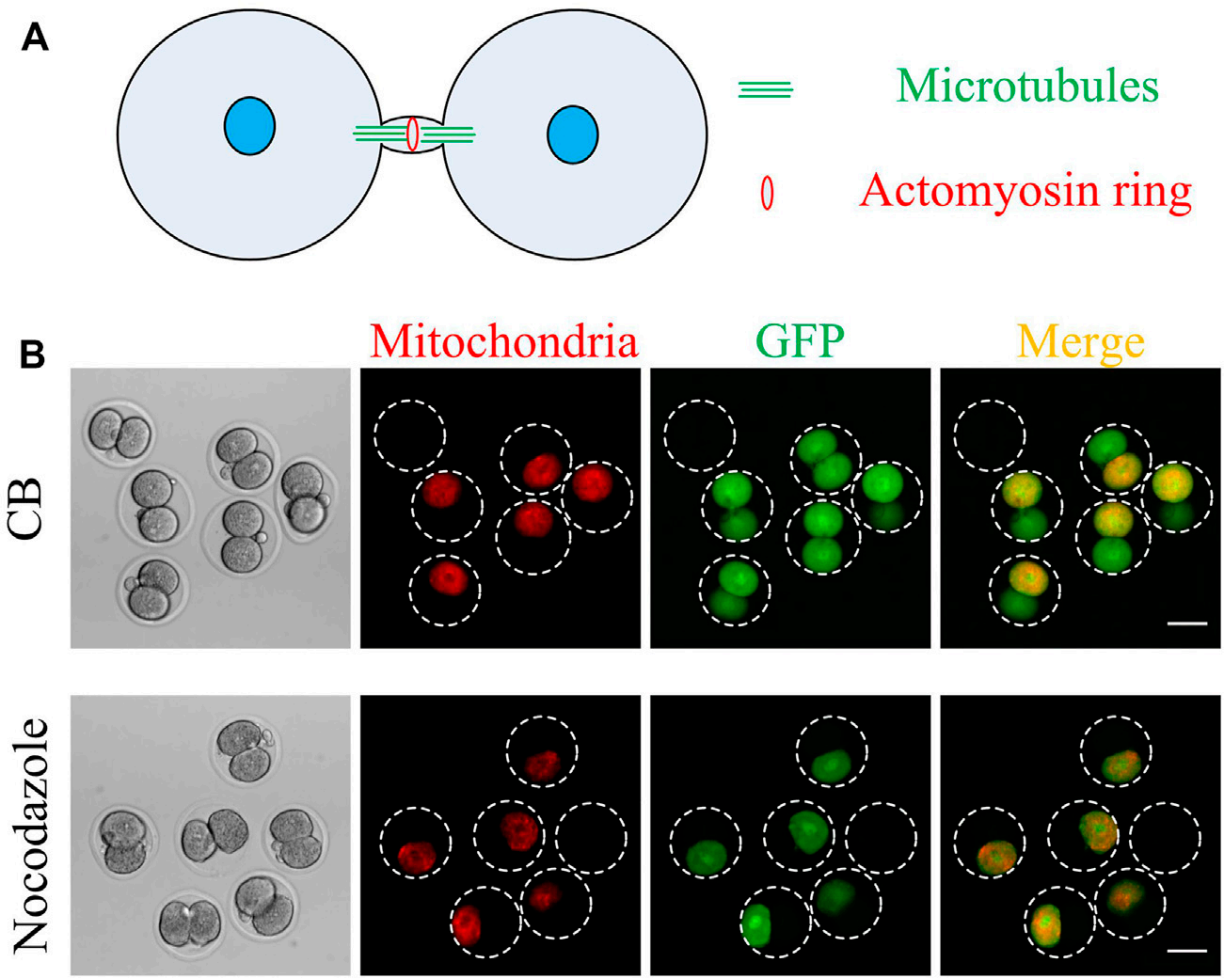
Nocodazole, but not cytochalasin B, inhibits cytoplasmic GFP transport between the two daughter cells. **(A)** A schematic of an intercellular bridge (modified from Chen, C. T. et al. Nature reviews. Molecular cell biology 2012, 13, 483–488). Microtubules and actomyosin rings are two of the most important components of the intercellular bridge. The microtubule is parallel, and the actomyosin ring is perpendicular to the intercellular bridge. **(B)** After 1 h of mouse embryo morphologically divided into two cells, the mRNA expressing GFP and mitochondrial localized RFP was microinjected into one daughter cell. Then the embryos were cultured with nocodazole or cytochalasin B for 7 h and then imaged. Scale bar = 50 μm.

As shown in Figure 4A, microtubules and microfilaments are the two most important components of the intercellular bridge (Chen et al., 2012). If cytoplasmic GFP transport depends on the intercellular bridge, then microtubules or microfilaments may play an essential role in it. Nocodazole and cytochalasin B were used to depolymerize the microtubules and microfilaments, respectively. Results shown in Figure 4B indicate that nocodazole, while not cytochalasin B, can stop the GFP transportation. Therefore, the microtubules rather than the microfilaments are responsible for intercellular transportation (Figure 4A).

## Discussion

Cytokinesis is the last step of cell division. It determines how much cytoplasm and organelles distribute into the daughter cells, which plays a vital role in cell differentiation and function (Li 2007). Thereby, understanding the first completion time of cytokinesis in mouse embryos should be helpful for people to study early embryo development.

After about 22 h of fertilization, the mouse zygotes morphologically divide as two daughter cells (Nagy et al., 2003). However, whether the cytokinesis is completed at the same time is unclear. By definition, the last step of cytokinesis is to abscise the intercellular bridge between the two daughter cells completely. Therefore, checking for the presence of intercellular bridges should be the gold standard for determining the completion time of cytokinesis. Microtubules are the essential components of an intercellular bridge. So the intercellular bridge could be observed by labelling the microtubules (Johnson and Maro 1985; Kidder et al., 1988; Chatzimeletiou et al., 2005). However, our results indicated that the intercellular bridges appeared only in a few 2-cell embryos but not in most of them (data not shown). The undetectable intercellular bridge may be from the fact that the diameter of 2-cell embryos is about 80 μm, which is beyond the depth scope of the fluorescence microscope. Moreover, in some 2-cell embryos, the intercellular bridge is too short to be observed. Therefore, we studied the possibility of determining the completion time of cytokinesis by monitoring the transportation of cytoplasmic proteins. Our results indicate that the cytoplasmic GFP, but not the mitochondria, could be transported from the mRNA-microinjected daughter cell to the other un-injected daughter cell. The first cytokinesis is completed about 15 h after the zygotes morphologically divide into two daughter cells. Moreover, this transportation is dependent on the microtubules while not microfilaments.

It is well known that intercellular bridges are stable and play a vital role in gametogenesis in multicellular animals. They connect the cytoplasm of adjacent germ cells to form syncytia and are large enough (0.5–3 μm) to allow most cytoplasm and even the organelles such as mitochondria to communicate with each other (Haglund et al., 2011). Unlike gametogenesis, the intercellular bridges in 2-cell mouse embryos allow only the cytoplasmic proteins, not mitochondria, to be passed through. The ratio of the GFP intensity between the two daughter cells in almost all of the embryos reached about 80%, which means that cytoplasmic protein transportation is highly effective and may play some important roles in embryo development.

Deep single-cell RNA-seq showed that some protein-coding genes showed uneven distribution in the two blastomeres of 2-cell mouse embryos, and thus, studies indicated that the two blastomeres might begin to differentiate as early as this time (Biase et al., 2014; Casser et al., 2019). However, it is well known that the two daughter cells of the 2-cell embryos are equally totipotent, and the cells during early embryos are highly synchronized (Casser et al., 2017; Wu et al., 2017). These results seem to be somewhat inconsistent. We supposed that the intercellular bridge might reverse the mRNA differences between the two daughter cells through cytoplasmic transportation in the 2-cell embryo, thus playing an essential role in these early developmental events. Therefore, 5 or 15 h after the embryos divided morphologically into 2-cells, they were bisected and reassembled, and the developmental potential and synchronization were examined. It seems that cytoplasmic protein communication has no significant effect on embryonic development and synchronization. We cannot rule out the possibility that the blastomeres may change their gene expression models, thereby responding to the termination of cytoplasmic protein transport.

In summary, in this study, we studied the dynamics of cytoplasmic protein transportation, which relies on microtubules, and demonstrated that the first completion time of cytokinesis is 15 h after the fertilized egg morphologically divides into a 2-cell embryo. Furthermore, our results indicate that the cytoplasmic protein transportation between the two blastomeres is highly effective without a significant effect on embryonic development and synchronization. This study may help people understand early embryo development. More studies should be conducted, such as whether an intercellular bridge is involved in the transportation of endogenous proteins between blastomeres, which can be helpful to rescue the low-quality blastomeres.

## Data Availability

The original contributions presented in the study are included in the article/Supplementary Material, further inquiries can be directed to the corresponding authors.
